# Abnormal Regional Homogeneity in Patients with Essential Tremor Revealed by Resting-State Functional MRI

**DOI:** 10.1371/journal.pone.0069199

**Published:** 2013-07-15

**Authors:** Weidong Fang, Fajin Lv, Tianyou Luo, Oumei Cheng, Wei Liao, Ke Sheng, Xuefeng Wang, Fei Wu, Yida Hu, Jing Luo, Qing X. Yang, Han Zhang

**Affiliations:** 1 Department of Radiology, the First Affiliated Hospital of Chongqing Medical University, Chongqing, China; 2 Department of Neurology, the First Affiliated Hospital of Chongqing Medical University, Chongqing, China; 3 Center for Cognition and Brain Disorders, Hangzhou Normal University, Hangzhou, China; 4 Zhejiang Key Laboratory for Research in Assessment of Cognitive Impairments, Hangzhou, China; 5 Center for NMR Research, Department of Radiology, Penn State College of Medicine, Hershey, Pennsylvania, United States of America; University of Minnesota, United States of America

## Abstract

Essential tremor (ET) is one of the most common movement disorders in human adults. It can be characterized as a progressive neurological disorder of which the most recognizable feature is a tremor of the arms or hands that is apparent during voluntary movements such as eating and writing. The pathology of ET remains unclear. Resting-state fMRI (RS-fMRI), as a non-invasive imaging technique, was employed to investigate abnormalities of functional connectivity in ET in the brain. Regional homogeneity (ReHo) was used as a metric of RS-fMRI to assess the local functional connectivity abnormality in ET with 20 ET patients and 20 age- and gender-matched healthy controls (HC). The ET group showed decreased ReHo in the anterior and posterior bilateral cerebellar lobes, the bilateral thalamus and the insular lobe, and increased ReHo in the bilateral prefrontal and parietal cortices, the left primary motor cortex and left supplementary motor area. The abnormal ReHo value of ET patients in the bilateral anterior cerebellar lobes and the right posterior cerebellar lobe were negatively correlated with the tremor severity score, while positively correlated with that in the left primary motor cortex. These findings suggest that the abnormality in cerebello-thalamo-cortical motor pathway is involved in tremor generation and propagation, which may be related to motor-related symptoms in ET patients. Meanwhile, the abnormality in the prefrontal and parietal regions may be associated with non-motor symptoms in ET. These findings suggest that the ReHo could be utilized for investigations of functional-pathological mechanism of ET.

## Introduction

Essential tremor (ET) is a progressive movement disorder found in human adults, with prevalence ranging from 4% in the population of age 40 years or older to 14% in those older than 65 years [1]. Despite its prevalence, there is little known concerning the functional pathology of ET [2]. Traditionally, ET has been regarded as a mono-motor symptomatic disease with postural or kinetic tremor(s) in the hands and forearms or isolated tremor(s) in the head and/or of the voice. Now, the evolving viewpoint regards ET as a heterogeneous neurologic disorder accompanied by several motor and non-motor symptoms [3]. The complicated clinical characteristics directed the ET origin to the central nervous system; most likely, in the cerebellum [4].

Consistent pathological markers for ET have not been identified because of limited postmortem studies owing to the low autopsy rate in ET patients. Several studies have shown that in patients with ET, 76% had a decrease in the number of cerebellar Purkinje cells and 24% had lower brainstem Lewy bodies [5]–[7]. However, Rajput and colleagues’ recent studies [8]–[10] showed that Purkinje cells and Lewy bodies were not the pathology of ET, which presented an entirely different picture.

Magnetic resonance imaging (MRI) has been employed to investigate ET in the literature. Recent MRI studies concentrated on structural (voxel-based morphometry or VBM) [11]–[15] and microstructural (diffusion tensor imaging or DTI) [16]–[19] abnormalities. Results from most studies [11]–[13], [17]–[19] have supported the hypothesis that the abnormalities of the cerebello-thalamo-cortical motor pathway and the fronto-parietal circuit are involved in the functional pathological changes of ET. However, several other studies showed that for most ET patients there was no cerebellar structural changes [15], and that there was no structural changes in above mentioned regions [14], [16]. In addition, these studies were lack of correlation between structural abnormalities and clinical symptom(s).

Task-designed fMRI is more specific to reflect the correlation between the clinical symptom(s) and brain dysfunction(s) [20]. Bucher and colleagues [21] reported abnormal activation of cerebellar networks involved in ET’s tremor generation by using imitation hand tremor stimulation. Other studies [22], [23] reported that the dysfunction of cerebello-prefrontal network and prefronto-parietal circuit were responsible for the non-motor symptoms in ET patients (through verbal working memory and stroop tasks). However, because ET patients always have difficulty in completion of task experiment, and the tremor by itself could introduce confounding factors (i.e., uncontrolled body motion), task-designed fMRI is not suitable for ET study. In addition, due to the lack of a unified stimulus paradigm, the existent task fMRI studies could not support each other, thus there is relatively little clinical use [24].

Resting-state fMRI (RS-fMRI) is a non-invasive technique that based on the spontaneous fluctuation of the blood-oxygen-level-dependent (BOLD) signal in fMRI [24], [25]. The RS-fMRI observes the brain’s functional change without requirement of an overt task performance or stimulation, thus avoids the inherent shortcomings of the task-designed fMRI [24], [26]. The RS-fMRI, as well as functional connectivity measurements derived from it, has been applied to the studies of brain dysfunction in movement disorders [27]–[29] and neurodegenerative diseases [30]–[32]. Unlike task fMRI which only focuses on single functional system at a time, the RS-fMRI provides brain functional connectivity or functional network information that will be valuable for simultaneously studying the mechanisms of motor and non-motor functional deficits in ET patients. Up to date to the best of our knowledge, only one paper [33] reported using independent component analysis (ICA) of RS-fMRI to explore the brain dysfunction in ET. In this study, ICA was applied to detect functional connectivity within the cerebello-thalamo-cortical network, where ET patients showed impaired functional connectivity. Although ICA is commonly used in RS-fMRI studies and has advantages as an exploratory tool over a hypothesis-based one (e.g., seed correlation), it also has inherent drawback of inconsistency problem induced by model order and initial value.

Regional homogeneity (ReHo) measurement of RS-fMRI provides an approach to investigate local functional connectivity based on temporal correlations between a voxel and adjacent voxels [34]–[35]. The ReHo analysis method has several advantages. First, it is based on data-driven approach and thus requires no prior knowledge [36], thus, it is more suitable for studying of a disease with unclear pathological mechanism such as ET. Second, the computation of ReHo using a Kendall’s coefficient of concordance is relatively simple and has been implemented in REST and DPARSFA software with many successful applications [37]–[40]. The biological meaning of the ReHo measurement has been proven by Zang and colleagues [35]. Recently, Zuo et al. [36] reported that the ReHo measurement has good test-retest reliability. Taken together, the ReHo method has been shown to be an excellent metric for quantification and detection of the functional pathological changes in neurologic disorders and neurodegenerative diseases [37]–[40]. However, to our knowledge, no report has applied ReHo in ET study.

The aim of this study was to quantify the abnormalities of local-range functional connectivity using ReHo on RS-fMRI in ET. For this purpose, we performed group comparison on ReHo maps between ET and healthy control (HC) groups. Second, several regions of interests (ROIs) were defined based on the group differences, followed by correlation analyses between the ReHo value in those ROIs and ET clinical assessments. Finally, an abnormal brain model with regards to various functional systems was proposed to link the brain dysfunctions with the ET symptoms in different dimensions.

## Materials and Methods

### Demographic and Clinical Evaluation

The diagnosis of ET was made according to the National Institute of Health Corroborative Genetic Criteria [41]. None of the patients had Parkinson’s disease (PD) or dystonia. Twenty ET patients (8 females; age: 50.3±14.2 years) and 20 gender- and age-matched HC subjects were included in our study. All subjects were right-handed and cognitively unimpaired without significant difference between the two groups (Mini Mental State Examination [42], or MMSE *>*24, *p* = 0.56). The mean age at onset of tremor was 35.3±9.9 years and mean duration was 14.6±7.7 years.

All patients had hand tremor(s), 2 patients with lower limb tremor(s), 5 patients with head tremor and 2 with voice tremor. A family history of tremors was present in 11 patients. For all the ET patients, this study was their initial visit, and before the RS-fMRI examination, all of them had no medication history. Alcohol ingestion history was positive for 2 patients and negative for 5 patients; the remain13 patients had never intoxicated but were unable to answer the question about alcohol to alleviate symptoms. The severity of tremor(s) was mild to severe, the mean TRS-A&B score was 21.1±14.7, and the mean TRS-C score was 6.9±4.9 (see Table 1 and Table S1).

**Table 1:** Demographic and clinical features of ET patients and HCs.

Measure	ET patients	Healthy controls	*P*-value
Demographic			
Age	50.3±14.2	50.3±14.2	1.00
Gender (male : female)	12∶8	12∶8	1.00
Handedness (right : left)	20∶0	20∶0	
Cognitive function			
MMSE	26.0±2.5	27.0±2.7	0.56
Clinical		NA	
Age of onset (years)	35.3±9.9	NA	
Disease duration (years)	14.6±7.7	NA	
Body parts with tremor		NA	
Upper limb	20 (20/20)	NA	
Lower limb	2 (2/20)	NA	
Head	5 (5/20)	NA	
Voice	2 (2/20)	NA	
Family history	11 (11/20)	NA	
Medication	None	NA	
Response to alcohol	2+ (2/20), 5– (5/20), 13 CAN (13/20)	NA	
Fahn-Tolosa-Marin Tremor Rating Scale (TRS)		NA	
TRS-A&B	21.1±14.7	NA	
TRS-C	6.9±4.9	NA	

ET: essential tremor, HCs: healthy controls, NA: not applicable, +: positive, −: negative, CAN: cannot answer.

All subjects recruited into the study gave their written informed consent approved by the Ethics Committee for the First Affiliated Hospital of Chongqing Medical University in accordance with the Declaration of Helsinki.

### Clinical Assessment

ET patients were evaluated by two movement disorder specialists (O Chen andxWang). Age at onset, disease duration, body parts with tremor, family history and, medication and alcohol usage in relation to alleviate symptoms were recorded. The severity of the tremor was assessed using Fahn-Tolosa-Marin Tremor Rating Scale (TRS) [43]. This scale is composed of three parts: TRS part A, B and C. The TRS part A and B were combined to obtain a single score: TRS-A&B. The TRS-A&B was used primarily in evaluation of tremor severity, location and, the drawing and writing function of hand. This was performed and recorded via videotape record and blinded to the RS-fMRI results. The TRS-C was assessed via self-evaluation to evaluate quality of life for ET patient.

### Image Acquisition

All MR images were acquired using a GE Signa Hdxt 3.0T scanner (General Electric Medical Systems, USA) with a standard 8-channel head coil. Foam padding was used to minimize the head motion and ear plugs were used to reduce scanner noise. Apart from this, no other special methods were employed to prevent head movement. During RS-fMRI acquisition, all subjects were told to relax, and keep still with eyes closed, but to remain awake (confirmed with post-scan debriefing).

RS-fMRI data were acquired using an echo-planar image (EPI) pulse sequence with 33 axial slices, thickness/gap = 4.0/0 mm, matrix = 64 × 64, TR = 2000 ms, TE = 40 ms, flip angle = 90°C, FOV = 240 × 240 mm. A total of 240 time points was obtained in 8 min.

High-resolution 3D-T1 (TR = 8.3 ms, TE = 3.3 ms, flip angle = 15°, thickness/gap = 1.0/0 mm, FOV = 240 × 240 mm, matrix = 256 × 192) and T2-FLAIR-weighted images (TR = 8000 ms, TE = 126 ms, TI = 1500 ms, thickness/gap = 5.0/1.5 mm, FOV = 240 × 240 mm, matrix = 256 × 192) were also acquired. We did not use T2-FLAIR-weighted images for data processing but for clinical evaluation and data quality assessment (see *Quality assurance*).

### Image pre-processing

Data pre-processing was conducted using the toolboxes of DPARSFA on version 2.1 (www.restfmri.net, [44]) and REST on version 1.8 (www.restfmri.net, [45]). It was mostly consistent with previous studies using ReHo [35], [38], [39], [46] and consisted of following steps briefly listed here: 1) removal of the first 10 time points, 2) slice timing correction, 3) realignment (for detailed discussion on head motion please see *Quality assurance*), 4) unified segmentation using 3D-T1 images and spatial normalization using the deformation parameters and, 5) time course de-trending and band-pass filtering (0.01−0.08 Hz) (see Text S1 for details). As described in previous studies [36], [39], spatial smoothing artificially enhances the ReHo intensity. This process was not performed during pre-processing. To improve Gaussianity for statistical analyses, the spatial smoothing was carried out after ReHo calculation. For observing small structures that were hypothesized to have abnormality in ET (e.g., ventral intermediate nucleus), we used a smaller smoothing kernel with FWHM of 4×4×4 mm^3^).

### Quality Assurance

The T1 and T2-FLAIR images were inspected by an experienced neuroradiologist (F Lv or T Luo). All subjects had no obvious abnormalities in gross brain structure. We noted that the head-motion is an important factor in RS-fMRI studies [36] and, especially, in the ET studies. To control the potential negative effect caused by excessive head motion (which may stronger for ET patients than HC), we adopted a strict criterion for head movements assessment (less than 1.0 mm and 1.0 degree in the *x*, *y* and *z* directions) and compared the maximal absolute head movement between the two groups. Despite having 5 patients with head tremors involved in our study, these tremors appeared as a postural and intentional. They did not resemble another tremor disease, such as Parkinson’s disease (PD patients have obvious resting-state tremors). Therefore, the head tremor was not expected to be a serious problem. All subjects’ head movement did not exceed the exclusion criteria, and the maximum absolute head movement showed no significant group difference (*p* = 0.35).

### ReHo Calculation and Statistical Analyses

Individual ReHo image was generated for each subject by calculating Kendall’s coefficient of concordance (KCC) at each voxel between the time series of this voxel and those of its 26 neighboring voxels within a whole-brain mask (this mask is provided by DPARSFA, excluding non-brain areas). Then, each subject’s ReHo image was standardized with regard to the global mean by dividing the ReHo value at each voxel by the averaged ReHo across the whole-brain (using the same whole brain mask). This global mean normalization method has been adopted as an ordinary processing procedure in previous ReHo studies [35], [38], [39], [46]. We also compared the global mean ReHo between two groups. No significant difference was found (two-sample *t* test, *p* = 0.82). The other reason we used this normalization method is to avoid spurious negative values that may be introduced by other methods (e.g., *z*-transformation), which are difficult to interpret.

One-sample *t* tests with AlphaSim multiple comparison correction was performed to compute the group-specific ReHo maps for each group, and to determine the brain regions with ReHo value significantly larger than the global mean ReHo. The brain regions with significant *t* values for both groups were combined to form a mask for a between-group comparison.

To detect group difference in ReHo, a two-sample *t*-test with AlphaSim multiple comparison correction was performed within the mask generated by the one-sample *t*-tests. Of note, the mean-corrected ReHo maps (i.e., standardized ReHo), rather than the raw ReHo maps were fed into the two-sample t test. Meanwhile, we also performed a two-sample *t*-test within the whole-brain mask to further explore the potential group difference in a pure data-driven manner.

### Clinical Correlation Analyses

Based on the two-sample *t*-test findings, the abnormal ReHo brain regions of ET were identified as ROIs. Then, the mean ReHo value in each ROI for all the subjects was extracted, and correlated by using Pearson’s correlation with the clinical assessment, including the TRS-A&B scores, the TRS-C scores, the age at onset and the disease duration. To improve data normality, the clinical scores were converted to *z* scores before the correlation analyses [42]. Kolmogorov-Smirnov tests were conducted to assess the normality of the *z* scores, showing good normality (*z*TRS-A&B: *Z* = 0.65, *p* = 0.78; *z*TRS-C: *Z* = 0.97, *p* = 0.30; *z*Age at onset: *Z* = 0.66, *p* = 0.76; *z*Disease duration: *Z* = 0.59, *p* = 0.86).

### VBM Analysis

To check out if there was structural difference between ET and HC, the high-resolution 3D-T1 images were subjected to VBM analysis using DPARSFA software. Briefly, the T1 images were segmented into grey matter (GM), whiter matter (WM) and cerebrospinal fluid (CSF) by using unified segmentation, and then normalized to MNI space. After that, two-sample *t*-tests with false discovery rate (FDR) corrections were performed to investigate group difference in GM and WM density.

## Results

### Within-group Analyses

**Figure 1:**
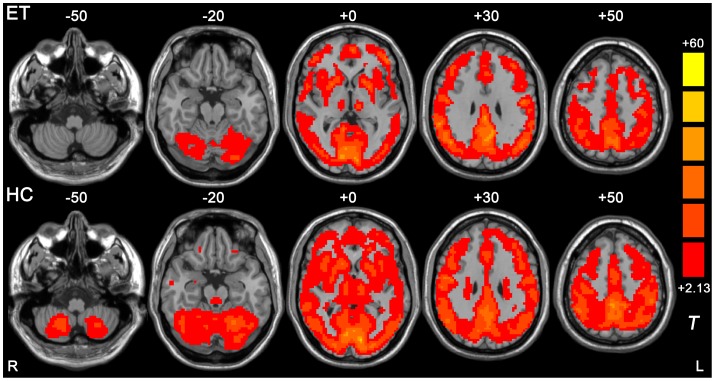
Results from one-sample *t*-test on ReHo maps for ET group (upper) and HC group (lower). Threshold was set to *p*<0.05 with AlphaSim correction (cluster size >2295 mm^3^). The underlying structure image is *Ch2* image.

The within-group ReHo t-maps for the ET and the HC group were shown in Figure 1. For both groups, extensive grey matter regions showed significant larger-than-global-mean ReHo values. Those regions include sensorimotor areas, visual areas, auditory areas, executive networks, prefrontal cortex, insula, middle and inferior temporal cortex, medial and lateral parietal cortex, cerebellum, striatum, thalamus and the default mode network. Compared with the HC group, the ET group showed extensive ReHo reductions, primarily located within the cerebellum and the thalamus.

### Group Difference Analyses

**Figure 2:**
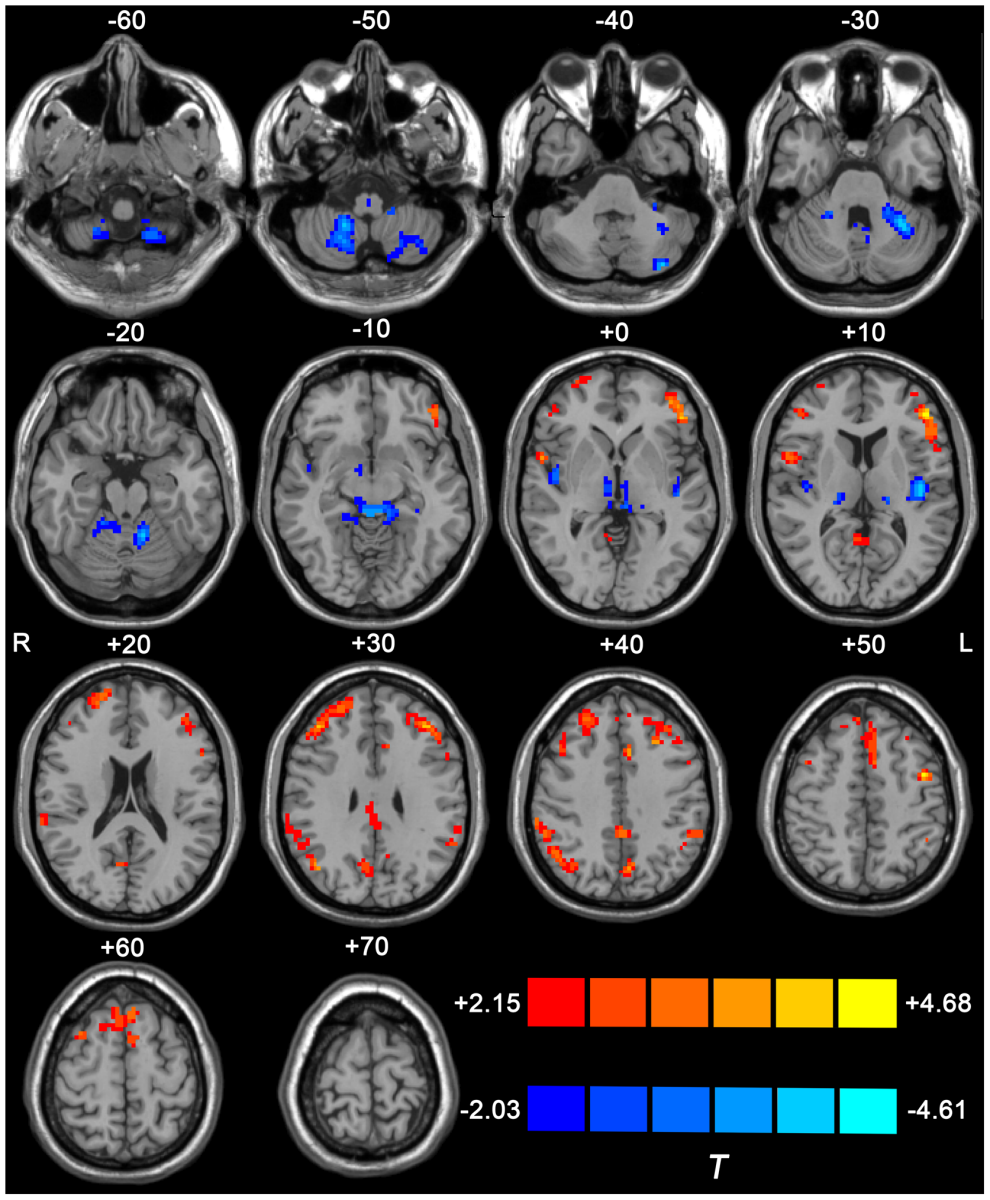
Difference in ReHo value between ET and HC groups. The result was derived from two-tailed two-sample *t* test within combined one-sample *t*-test mask (*p*<0.05, AlphaSim corrected with cluster size >351 mm^3^). Warm color indicates the regions with larger ReHo value in ET than HC, while cold color indicates those with decreased ReHo in ET. The underlying structure image is *Ch2* image.

Figure 2 shows the group difference in ReHo within one-sample *t*-test mask between the two groups. Significant ReHo decrease was found in extensive cerebellar cortices, including the anterior (lobules III∼V) and posterior (bilateral lobules VIII and left VI) bilateral cerebellar lobes, the posterior insular lobes and the bilateral thalamus which included mediodorsal (MD) and ventral intermediate (VIM) thalamic nucleus. A significant ReHo increase were found in the left primary motor cortex, the left supplementary motor area, the bilateral prefrontal cortices (including dorso- and ventro-lateral prefrontal cortex, orbitofrontal cortex, and the medial prefrontal cortex) and the bilateral parietal lobes (including the angular gyrus, the supramarginal gyrus and the inferior parietal lobule). The details of the peak coordinates, cluster size and Brodmann’s areas are listed in Table 2. Meanwhile, the group difference results found within the whole brain mask are shown in Figure S1 and Table S2. The two results with different masks were quite similar.

**Table 2:** Differences in ReHo between ET patients and HCs.

Brain region	MNI coordinates			*t* value	cluster size (voxels)
	x	y	z		
R Cerebellum VIII	20	–55	–60	–2.97	83
L Cerebellum VIII	–33	–58	–45	–2.78	96
L Cerebellum VI	–30	–50	–30	–3.92	86
L Cerebellum IV, V	–24	–41	–30	–3.45	88
L Cerebellum III	–7	–38	–15	–2.91	22
R Cerebellum IV, V	14	–43	–15	–3.25	55
R Cerebellum III	10	–38	–15	–2.91	18
L Brainstem (inferior olivary nucleus)	–3	–40	–45	–3.26	18
R Brainstem (inferior olivary nucleus)	4	–40	–45	–3.08	21
R Thalamus (ventral intermediate, VIM)	16	–22	0	–2.86	23
L Thalamus (VIM)	–14	–20	0	–3.12	21
R Thalamus (mediodorsal, MD)	6	–12	0	–2.91	24
L Thalamus (MD)	–5	–17	0	–2.45	26
L Insula	–41	–13	15	–3.16	49
R Insula	44	–6	0	–3.23	21
R Superior frontal gyrus orbital part	28	64	0	2.42	28
R Inferior frontal gyrus triangular part	44	35	15	2.80	32
R Middle frontal gyrus	38	37	30	4.05	41
R Superior frontal gyrus	18	52	30	2.58	27
L Superior frontal gyrus orbital part	–45	37	–15	3.76	43
L Middle frontal orbital part	–40	48	0	3.38	51
L Inferior frontal gyrus triangular part	–50	36	0	3.55	19
L Middle frontal gyrus	–40	37	30	4.01	37
L Supplementary motor area	–6	14	45	4.09	52
R Supplementary motor area	4	18	60	3.12	56
L Precentral gyrus	–49	–1	45	3.45	43
R Supramarginal gyrus	64	–41	30	2.89	37
L Supramarginal gyrus	–61	–40	30	2.89	29
R Inferior parietal gyrus	46	–57	45	3.15	19
L Inferior parietal gyrus	–46	–50	45	2.34	29
R Angular gyrus	51	–51	30	2.86	21

ET: essential tremor, HCs: healthy controls, R: right, L: left.

### Correlation Analyses

**Figure 3:**
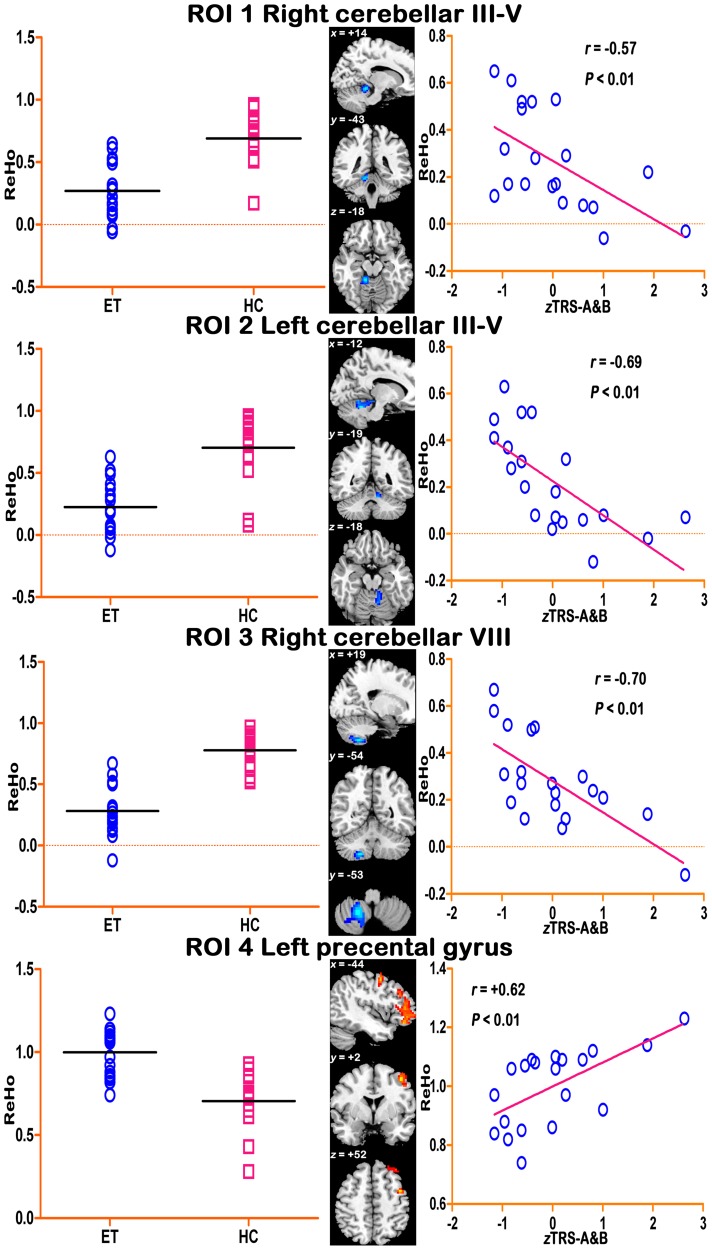
ROIs with abnormal ReHo in ET group compared with HCs and the correlation between mean ReHo values in each ROI and TRS-A&B scores. The left panel shows the average ReHo value in each ROI for subjects in both ET and HC groups. The middle panel shows the location of the ROIs. The right panel shows the scattered maps of the mean ReHo against the TRS-A&B scores in regions with significant (*p*<0.01, uncorrected) correlations. TRS-A&B: the combination between the scores of the Fahn-Tolosa-Marin Tremor Rating Scale (TRS) part A and B.

There were 24 clusters which showed significantly abnormal ReHo values in ET compared with HC groups, which were further defined by 24 ROIs. Correlation analyses for each ROI revealed 4 ROIs with significant correlations with the *z*TRS-A&B scores in the ET group. For other ROIs and other clinical scores including the TRS-C scores, the age at onset and the disease duration, the correlations were non-significant. Specifically, a significantly negative correlation was found in the bilateral cerebellar lobules III∼V (peak MNI coordinates for the left one: *x* = −12, *y* = −19, *z* = −18, *r* = −0.69, *p*<0.01; the right one: *x* = +14, *y* = −43, *z* = −18, *r* = −0.57, *p*<0.01, both uncorrected) and the right cerebellar lobule VIII (*x* = +19, *y* = −54, *z* = −53, *r* = −0.70, *p*<0.01, uncorrected). Significant positive correlation was observed in the left precentral cortex (*x* = −44, *y* = +2, *z* = +52, *r* = 0.62, *p*<0.01, uncorrected) (For the location of these ROIs, box and scatter plots, please see Figure 3.) Please note that, because of the relatively small group size and the exploratory researching methods, we used an uncorrected *p-*value of 0.01 (rather than a stringent multiple comparison correction method, e.g., Bonferroni correction).

### VBM Analysis

The VBM analysis did not detect any significant differences in either grey or white matter density between our study groups. This was consistent with part of the previous studies [14], [15], [17]. Therefore, grey or white matter density may not potentially affect the RS-fMRI result, and thus was not taken into account as nuisance covariables in the two-sample *t*-test.

## Discussion

We used the ReHo metric of RS-fMRI to reveal the local functional connectivity abnormality in ET patients compared with HC. We found that the ReHo abnormality was mostly located in the cerebello-thalamo-cortical motor pathway and non-motor cortices (including prefrontal, parietal and insular lobes). We propose that the disruption of local functional connectivity in the cerebello-thalamo-cortical motor pathway is involved in tremor generation and propagation of ET. Conversely, the abnormal ReHo in the prefrontal, parietal and insular lobes may take responsibility for the non-motor symptoms (e.g., cognitive, psychiatric and sensory deficits). The VBM analysis did not detect significant grey or white matter atrophy between our study groups. Each of those abnormal brain regions we found will be discussed respectively in the following sections.

### Cerebellum

There is a constant debate on whether the cerebellum acts as a tremor generation structure or not in ET. On the one hand, there is a bulk of evidence from histopathological [5], electrophysiological [47], neuroimaging [4] and treatment effect [33] which support that the cerebellum plays a crucial role in ET. However, opposing views are constantly reported [8], [10], [16]. Our study, which found that extensive cerebellar ReHo abnormalities exist in ET, further lends support to the evidence and reinforces the point of the major role of the cerebellum in ET.

First, we found that cerebellar ReHo abnormality was not only located within the spino-cerebellum (the bilateral anterior cerebellar lobules III∼V), but also extended to the cerebro-cerebellum (the bilateral posterior cerebellar lobules VIII and the left lobule VI). It appears that the extensive ReHo abnormalities in the cerebellum were not associated merely with ET pathophysiological basis. As mentioned in the *Introduction* section, ET was typically seen as a mono-motor symptomatic disease, with only superior part of the cerebellum was involved. However, the evolving concepts now regard ET as heterogeneous disorders accompanied by a lot of movement and non-motor symptoms [3], [48], [49]. Several lines of evidence from clinical [50]–[54], neuroimaging [55], [56] and functional topography of the cerebellum [57], [58] supported our finding that the extensive cerebellar ReHo abnormalities are involved in pathological changes of ET. First, clinical studies supported the cerebellar ReHo abnormalities involved in these movement and non-motor symptoms. Despite this, tremors are the cardinal clinical features of ET. There are multiple-component motor and non-motor dysfunctions in ET. Balance and gait disorder [52], [59], ataxia and eye-blink conditioning deficits [60], [61] was associated with spinocerebellar impairment. Impaired reaching and hand function [62] suggests spinocerebellar and cerebrocerebellar dysfunctions. Working memory deficits, cognitive disorder, personality disorder and social dysfunction [3], [53] are also related to cerebrocerebellar dysfunction. The cerebellar ReHo abnormalities in our study offer a plausible explanation to support the notion that the extensive cerebellar dysfunction is associated with the heterogeneous symptoms in ET. Secondly, neuroimaging studies support the extensive cerebellar dysfunction linked with ET diverse symptoms. Evidence from task-designed fMRI [21]–[23] and Positron Emission Tomography (PET) [63] showed that the abnormal activations in different cerebellar regions while ET patients were performing motor and non-motor tasks. These cerebellar areas were consistent with our result. In contrast to the task-based approach which requires on complex task stimulus, the RS-fMRI method provides an approach to identify simultaneously the cerebellar motor and non-motor networks using the same dataset without any stimulus. Therefore, the RS-fMRI technique is more suitable for observing these network changes in the pathological state of ET in the clinical realm. Finally, evidence from the functional topography of the cerebellum [57], [58] supported the finding that the widely cerebellar ReHo abnormalities caused heterogeneous disorders in ET. The different region of the cerebellum is associated with different physiological functions. Motor and non-motor task-designed fMRI studies showed the finger-taping stimuli often activated the anterior and posterior cerebellar lobes [57], [58]; and a non-movement stimulus (such as language, spatial processing and working memory tasks) often activated the posterior cerebellar lobe [57], [58]. Taken together, we speculated that ReHo abnormalities in these areas may be a hallmark of physiological dysfunction, which later appears as a heterogeneous disorder in ET. In application, our study provided more targets for cerebellar repetitive Transcranial Magnetic Stimulation (rTMS) for ET treatment. We propose that applying rTMS on our derived cerebellar regions with abnormal ReHo values, rather than traditionally only on the anterior or posterior cerebellar lobes, could largely improve patient’s quality of life by reducing the tremor symptom while restoring the impaired high-order functions.

Secondly, we found a different result in ET when compared with another tremor disease, PD. Literature [28] has reported that ET and PD shared similar clinical manifestations and a tremor-generation central network. Wu and colleagues [38] also used the RS-fMRI method and showed that PD patients had increased ReHo values in the very cerebellar regions that we reported, although our ReHo values were decreased. We postulated that such a difference may reflect the pathological difference between the two diseases. It has been observed that the loss of cerebellar Purkinje cells probably involved in the generation of tremors in ET [5]. The Purkinje cells are the only efferent neurons in cerebellum, which coordinate the function between the cerebellar cortices and the deep cerebellar nucleus [64]. While it was damaged, the local concordance and connectivity would decrease and lead to a deceased ReHo value. However, no pathological lesions in the cerebellum have been found in PD, and the PD tremors were believed to be mainly driven by the dysfunction of the globus pallidus loops [65]. We proposed that, in contrast to cerebellar ReHo increase which may be a compensation mechanism in PD [38], the cerebellar ReHo decrease in ET maybe a direct result of the cerebellar pathological damage.

### Prefrontal/Parietal/Insular Cortices

Interestingly, our study found that extensive ReHo abnormalities in the non-motor cortices, including the prefrontal, parietal cortices and the posterior insular lobes. These brain regions did not involve in the tremor symptom [4], but were included in the cognitive control network, default mode network and affective networks [66]. Evidences from task fMRI [67] and RS-fMRI [68] have shown that dysfunctions in these networks may result in various non-motor disorders, including cognitive deficits, dementia, anxiety, depression and sleeping deficit. These may explain why ET patients have heterogeneous non-motor symptoms. Meanwhile, epidemiological research [69] has revealed that the risk of developing dementia in patients with ET was significantly higher. This indicated that ET patients may share the similar cognitive impairment with AD patients. Recent RS-fMRI studies have revealed that ReHo was decreased in extensive brain areas in AD patients, including prefrontal, parietal cortices and default mode network. We also observed ReHo changed in the similar areas, but our findings were increased ReHo values, and ET patients in our study had no dementia, as confirmed by MMSE scores [70]. Therefore, we proposed that ReHo abnormalities in these brain areas may serve as a compensatory mechanism which prevents ET patients from apparent dementia symptoms. At the same time, ET patients already have potential cognitive dysfunction.

### Thalamus

Our study found that two thalamus regions, VIM and MD, showed abnormal ReHo values. To the best of our knowledge, this is the first report that the spontaneous brain activity abnormality existed in the thalamus of ET patients. Studies [28], [71] have shown that there are functional and structural connections between thalamus, cerebellum and the cortices. The VIM (local maxima of group difference was at [−14, −20, 0] in MNI space for the left, and [+16, −22, 0] for the right) connects cerebello-VIM-motor pathway, through which the cerebellum modulates motor function. This pathway has been proposed to be responsible for tremor propagation in ET patients [28]. The MD (local maxima was at [−5, −17, 0] for the left, and [+6, −12, 0] for the right) is another large nucleus in thalamus, and connects the prefrontal cortices, limbic system and cerebellum, through which the cerebellum modulates non-motor function [72]. Our study indicated that thalamus is not only involves in the motor dysfunction but also in the non-motor dysfunctions. In future, specific cognitive evaluation will help to understand its mechanism.

### Motor Cortex

The motor cortex is involved in generation of pathological tremor in ET, PD and cortical myoclonus patients [73]. However, the way of its involvement differs between the cortical myoclonus and ET or PD. Cortical myoclonus originates directly from primary motor cortex (M1) and is related to the hyper-excitability of the primary motor areas. In contrast, tremors in ET or PD arise from subcortical structures, i.e., the cerebello-thalamic circuit [65]. The motor cortex is considered to only serve as a mediated cortex of the oscillations. Therefore, the motor cortex should not be regarded as a substantial tremor generation site. As a result, we proposed the ReHo abnormality in the motor cortex might just be a direct consequence of the functional abnormality in the cerebello-thalamic pathway.

### Inferior Olivary Nucleus

Animal studies have demonstrated the inferior olivary nucleus is involved in tremor generation for ET [74]. Our finding of decreased ReHo in the inferior olivary nucleus was consistent with previous reports [74]. It must be noted that the spatial resolution of the fMRI in our experiment was low, and abnormality in such a small brain structure should be interpreted with caution.

### Correlation Analyses

Our study showed the ReHo abnormalities in the cerebellum and left primary motor cortex correlated with the tremor severity score in ET patients. Previous studies [73], [75] showed that these areas are involved in voluntary and involuntary body movement, and dysfunction in these areas will lead to pathological tremors. Therefore, our correlation analyses provide additional evidence that abnormal ReHo in cerebellum and motor-related cortices are involved in the tremor generation and propagation for ET. Meanwhile, no significant correlation was found between the mean abnormal ReHo values in other brain areas and clinical assessment, including TRS-C score, age at onset and disease duration. Perhaps an explanation for this was the reliability of the TRS-C score was low (based on self report), or the ReHo metric was not sensitive to these clinical assessments.

### Limitations

We found extensive brain dysfunctions reflected by altered ReHo, which indicated pathological changes of ET. However, we could not conclude that ET is a neurodegenerative disease merely based on present findings due to this study is cross-sectional by nature and the absence of correlation with disease duration. In future, a longitudinal or follow-up study with more homogenous samples would help to fully understand this feature. Meanwhile, despite abnormal ReHo found in the cerebello-thalamo-cortical motor pathway and non-motor cortices, the relationship between these nodes cannot be revealed using the current method. The two regions may have some relationships. However, such relationships might be caused by a third region or other reasons. The correlation between the ReHo changes in frontal lobe and those in cerebellum should be interpreted with caution. For example, we cannot decide whether the frontal lobe dysfunction was originated from the malfunctioned cerebellum or was just part of the extensive dysfunctions in ET. A long-range functional connectivity or a DTI study should be done in future.

### Methodological/Reliability Concerns

The pre-processing (and post-processing) procedure is an important methodological issue for RS-fMRI studies. No consensus has been obtained in the literature [36]. However, this factor plays an important role in the correctness and reliability of the results. Few studies have investigated the test-retest (TRT) reliability of different derivatives from resting-state fMRI, such as seed-based functional connectivity [76], ICA [77] and amplitude of low frequency fluctuations (ALFF) [78]. Meanwhile, there is already a pioneering study [36] systemically investigating these factors and their contributions to the TRT reliability of ReHo. The study recommended regressing out head motion, white matter and CSF signals, but not removal of the global signal in ReHo computation [36]. The study also found that the spatial smoothing will significantly influent the result reliability [36]. The study [36] also compared different head motion correction methods and found there was no significant difference on the TRT reliability between a traditional 6-parameter rigid transformation and newly proposed micro-head motion correction [79]–[82]. Since our study mainly deals with a brain disease, we decided not to systematically compare the results between various pre-processing methods, which include nuisance regressing, micro-head motion and the size of the spatial smoothing kernel. Another reason we did not do this is because we wanted our results to be compared with previous PD study [38] using the same data processing method.

### Conclusions

Using ReHo of RS-fMRI as a metric, we revealed the local functional connectivity abnormality in ET patients compared with HCs. These abnormalities were mostly located in the cerebello-thalamo-cortical motor pathway and non-motor cortices, including the prefrontal, parietal and insular lobes. Our analyses indicated that the ReHo abnormalities in different regions were associated specifically with motor and non-motor symptoms of ET. We demonstrated that our approach can be used to detect and quantify the functional pathological changes in ET.

## Supporting Information

Figure S1 **Difference in ReHo value between ET and HC groups within a whole brain mask.** Threshold was set to be *p*<0.05 with AlphaSim correction. Warm color indicates the regions with larger ReHo value in ET than HC, while cold color indicates those with decreased ReHo in ET. The underlying structure image is *Ch2* image.
(TIF)

Table S1 **Detailed demographic and clinical features of ET patients.**
(DOCX)

Table S2 **The brain areas with group differences in ReHo value between ET and HC groups within a whole brain mask.**
(DOCX)

Text S1 **The detailed data preprocessing steps.**
(DOCX)
