# Supplementary figures and images for: Abnormal Regional Homogeneity in Patients with Essential Tremor Revealed by Resting-State Functional MRI
Source: PLoS One. 2013 Jul 15;8(7):e69199. doi: 10.1371/journal.pone.0069199 (PMC3711903; doi:10.1371/journal.pone.0069199)

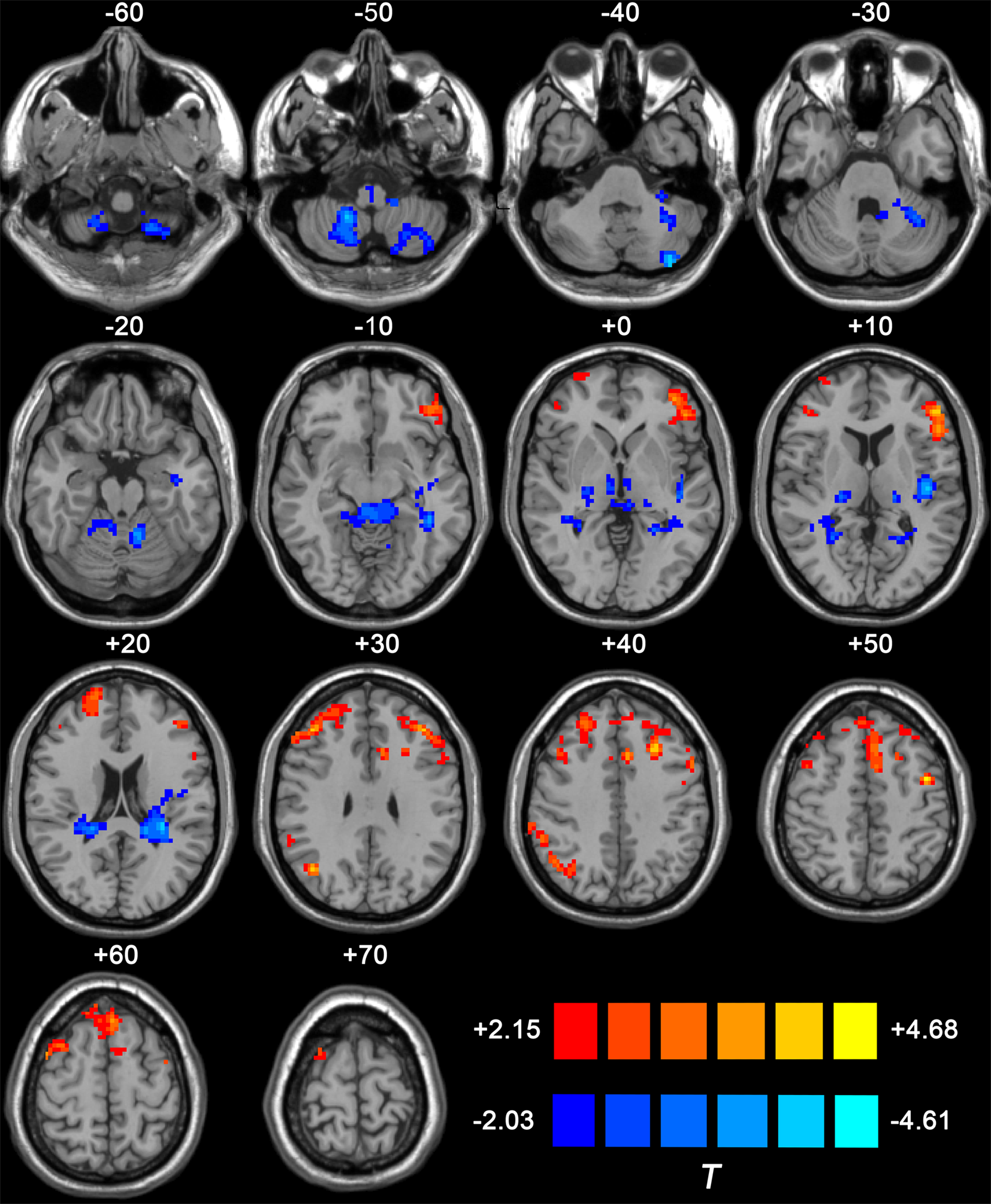

Supplement: Figure S1 — Difference in ReHo value between ET and HC groups within a whole brain mask. Threshold was set to be p<0.05 with AlphaSim correction. Warm color indicates the regions with larger ReHo value in ET than HC, while cold color indicates those with decreased ReHo in ET. The underlying structure image is Ch2 image. (TIF) [file pone.0069199.s001.tif]
