# Supplementary material for: Abnormal Regional Homogeneity in Patients with Essential Tremor Revealed by Resting-State Functional MRI
Source: PLoS One. 2013 Jul 15;8(7):e69199. doi: 10.1371/journal.pone.0069199 (PMC3711903; doi:10.1371/journal.pone.0069199)
Supplement: Table S1 — Detailed demographic and clinical features of ET patients. (DOCX) [file pone.0069199.s002.docx]

**Table S1.** Detailed demographic and clinical features of ET patients.

| No | Age | Sex | FH | Duration  (years) | Response  to alcohol | Medication | Tremor parts | | | | TRS | |
| --- | --- | --- | --- | --- | --- | --- | --- | --- | --- | --- | --- | --- |
|  |  |  |  |  |  |  | UL | LL | H | V | A&B | C |
| 1 | 28 | M | + | 7 | - | None | R=L | - | - | - | 22 | 15 |
| 2 | 56 | M | + | 9 | + | None | R=L | R=L | + | +  = | 60 | 21 |
| 3 | 51 | M | + | 24 | CNA | None | R=L | - | - | - | 13 | 6 |
| 4 | 57 | F | + | 34 | - | None | R=L | - | - | - | 21 | 4 |
| 5 | 65 | M | + | 18 | CNA | None | R=L | - | - | - | 24 | 6 |
| 6 | 58 | M | + | 14 | CNA | None | R=L | R=L | + | + | 49 | 14 |
| 7 | 29 | M | + | 7 | - | None | R=L | - | - | - | 9 | 3 |
| 8 | 68 | M | - | 24 | + | None | R=L | - | + | - | 16 | 8 |
| 9 | 30 | F | - | 5 | CNA | None | R=L | - | - | - | 4 | 0 |
| 10 | 55 | M | + | 14 | CNA | None | R=L | - | - | - | 25 | 8 |
| 11 | 43 | F | - | 9 | CNA | None | R=L | - | - | - | 30 | 7 |
| 12 | 27 | M | - | 6 | - | None | R=L | - | - | - | 22 | 8 |
| 13 | 64 | F | + | 20 | CNA | None | R=L | - | - | - | 12 | 2 |
| 14 | 58 | F | - | 18 | CNA | None | R=L | - | + | - | 7 | 3 |
| 15 | 49 | M | - | 13 | - | None | R=L | - | - | - | 8 | 3 |
| 16 | 64 | F | - | 21 | CNA | None | R=L | - | + | - | 12 | 5 |
| 17 | 77 | F | + | 22 | CNA | None | R<L | - | - | - | 36 | 6 |
| 18 | 36 | F | + | 11 | CNA | None | R=L | - | - | - | 15 | 7 |
| 19 | 52 | M | - | 10 | CNA | None | R=L | - | - | - | 4 | 10 |
| 20 | 44 | M | - | 7 | CNA | None | R>L | - | - | - | 33 | 3 |

M: Male, F: Female, FH: Family history, +: Postive or present, -: Negative or absent, Duration: duration of ET, CNA: cannot answer, UL: Upper limb, LL: Lower limb, L: Left, R: Right, H: Head, V: Voice, TRS: Fahn-Tolosa-Marin Tremor Rating Scale, A&B: TRS part A and B, C: TRS part C.
