# Supplementary material for: Abnormal Regional Homogeneity in Patients with Essential Tremor Revealed by Resting-State Functional MRI
Source: PLoS One. 2013 Jul 15;8(7):e69199. doi: 10.1371/journal.pone.0069199 (PMC3711903; doi:10.1371/journal.pone.0069199)
Supplement: Table S2 — The brain areas with group differences in ReHo value between ET and HC groups within a whole brain mask. (DOCX) [file pone.0069199.s003.docx]

**Table S2.** The brain areas with group differences in ReHo value between ET and HC groups within a whole brain mask.

| Brain region | MNI coordinates | | | *t* value | cluster size (voxels) |
| --- | --- | --- | --- | --- | --- |
|  | x | y | z |  |  |
| R Cerebellum VIII | 20 | -55 | -60 | -2.97 | 134 |
| L Cerebellum VIII | -33 | -58 | -45 | -2.78 | 101 |
| L Cerebellum VI | -30 | -50 | -30 | -3.92 | 90 |
| L Cerebellum IV, V | -24 | -41 | -30 | -3.45 | 100 |
| L Cerebellum III | -7 | -38 | -15 | -2.91 | 87 |
| R Cerebellum IV, V | 14 | -43 | -15 | -3.25 | 95 |
| R Cerebellum III | 10 | -38 | -15 | -2.91 | 85 |
| L Brainstem (inferior olivary nucleus) | -3 | -40 | -45 | -3.26 | 94 |
| R Brainstem (inferior olivary nucleus) | 4 | -40 | -45 | -3.08 | 112 |
| R Thalamus (ventral intermediate, VIM) | 16 | -22 | 0 | -2.66 | 103 |
| L Thalamus (VIM) | -14 | -20 | 0 | -3.06 | 91 |
| R Thalamus (mediodorsal, MD) | 6 | -12 | 0 | -3.16 | 87 |
| L Thalamus (MD) | -5 | -17 | 0 | 2.42 | 88 |
| L Insula | -41 | -13 | 15 | 2.80 | 91 |
| R Insula | 44 | -6 | 0 | 4..05 | 96 |
| R Superior frontal gyrus orbital part | 28 | 64 | 0 | 2.58 | 95 |
| R Inferior frontal gyrus triangular part | 44 | 35 | 15 | 3.76 | 87 |
| R Middle frontal gyrus | 38 | 37 | 30 | 3.38 | 96 |
| R Superior frontal gyrus | 18 | 52 | 30 | 3.55 | 94 |
| L Superior frontal gyrus orbital part | -45 | 37 | -15 | 4.01 | 85 |
| L Middle frontal orbital part | -40 | 48 | 0 | 4.09 | 103 |
| L Inferior frontal gyrus triangular part | -50 | 36 | 0 | 3.12 | 115 |
| L Middle frontal gyrus | -40 | 37 | 30 | 3.45 | 112 |
| L Supplementary motor area | -6 | 14 | 45 | 2.89 | 103 |
| R Supplementary motor area | 4 | 18 | 60 | 2.89 | 85 |
| L Precentral gyrus | -49 | -1 | 45 | 3.49 | 91 |
| R Supramarginal gyrus | 64 | -41 | 30 | 3.15 | 94 |
| L Supramarginal gyrus | -61 | -40 | 30 | 2.34 | 86 |
| R Inferior parietal gyrus | 46 | -57 | 45 | 2.86 | 85 |
| L Inferior parietal gyrus | -46 | -50 | 45 | -2.97 | 134 |
| R Angular gyrus | 51 | -51 | 30 | -2.78 | 101 |
