# Supplementary material for: Abnormal Regional Homogeneity in Patients with Essential Tremor Revealed by Resting-State Functional MRI
Source: PLoS One. 2013 Jul 15;8(7):e69199. doi: 10.1371/journal.pone.0069199 (PMC3711903; doi:10.1371/journal.pone.0069199)
Supplement: Text S1 — The detailed data preprocessing steps. (DOCX) [file pone.0069199.s004.docx]

**The detailed data preprocessing steps**

The data preprocessing consisted of the following steps:

1. *Removal of the first 10 time points*. For the purposes of scanner stabilization and the subjects’ acclimating to the MR scanning environment, the first 10 volumes were discarded, remaining 230 time points.

2. *Slice timing correction.* This was used to correct for different acquisition time across slices.

3. *Realignment.* This was used to realign the functional brain images to the first volume to correct for within-run head motions, resulting in six rigid-body head motion parameters. These parameters were employed to assess head movement and insure the quality of RS-fMRI data.

4. *T1 segmentation and spatial normalization.* 3D T1-weighted images were segmented into grey matter (GM), white matter (WM), and cerebrospinal fluid (CSF) probability maps using SPM unified segmentation. All the GM, WM and CSF images were resampled to 3 × 3 × 3 mm^3^ and, after that, spatially normalized to the MNI space using both affine transformation and non-linear deformation. The deformation field was applied to the RS-fMRI data (before step 4, the 3D-T1 images were co-registered to the mean RS-fMRI data for each subject).

5. *Smoothing.* As previous studies described, the spatial smoothing will artificially enhance the ReHo intensity, we did not perform it during data preprocessing. To improve Gaussianity for statistical analysis, the spatial smoothing was carried out after calculation of the individual ReHo maps. For observing small structures that were hypothesized to have abnormality in ET such as the ventral intermediate nucleus (VIM), we used a smaller smoothing kernel (FWHM: 4×4×4 mm^3^).

6. *Detrending and filtering.* These steps removed the extremely low frequency drift and the high frequency physiological noises. For detrending we used 1^st^ order polynomial functions and, for filtering, we adopted band-pass filtering (0.01 Hz < *f* < 0.08 Hz) to the time series for each voxel.
